# Caffeine on the mind: EEG and cardiovascular signatures of cortical arousal revealed by wearable sensors and machine learning—a pilot study on a male group

**DOI:** 10.3389/fnsys.2025.1611293

**Published:** 2025-09-15

**Authors:** Shabbir Chowdhury, Ahmed Munis Alanazi, Eyad Talal Attar

**Affiliations:** ^1^Department of Electrical and Computer Engineering, Faculty of Engineering, King Abdulaziz University, Jeddah, Saudi Arabia; ^2^Center of Excellence in Intelligent Engineering Systems (CEIES), King Abdulaziz University, Jeddah, Saudi Arabia

**Keywords:** caffeine, EEG, heart rate variability, blood pressure, cortical arousal, beta waves, machine learning, wearable sensors

## Abstract

**Introduction:**

Caffeine is the most widely consumed psychoactive substance, and its stimulant properties are well documented, but few investigations have examined its acute effects on brain and cardiovascular responses during cognitively demanding tasks under ecologically valid conditions.

**Method:**

This study used wearable biosensors and machine learning analysis to evaluate the effects of moderate caffeine (162 mg) on heart rate variability (HRV), entropy, pulse transit time (PTT), blood pressure, and EEG activity. Twelve healthy male participants (20–30 years) completed a within-subjects protocol with pre-caffeine and post-caffeine sessions. EEG was recorded from seven central electrodes (C3, Cz, C4, CP1, CP2, CP5, CP6) using the EMOTIV EPOC Flex system, and heart rate (HR) and blood pressure (BP) were continuously monitored via the Huawei Watch D. Data analysis included power spectral density (PSD) estimation, FOOOF decomposition, and unsupervised k-means clustering.

**Results:**

Paired-sample t-tests assessed physiological and EEG changes. Although systolic and diastolic BP showed a non-significant upward trend, HR decreased significantly after caffeine intake (77 ± 5.3 bpm to 72 ± 2.5 bpm, *p* = 0.027). There was a significant increase in absolute alpha power suppression (from −5.1 ± 0.8 dB to −6.9 ± 0.9 dB, *p* = 0.04) and beta power enhancement (−4.7 ± 1.2 dB to −2.3 ± 1/1, *p* = 0.04). The surface data from FOOOF shows these are real oscillatory changes. Based on the changes in clustering prior and post-caffeine, a machine-learning change in the brain activity differentiated pre/post-caffeine states with unsupervised clustering. The study results show that moderate caffeine resulted in synchronized EEG and cardiovascular changes, indicating increased arousal and cortical activation that are detectable with wearable biosensors and classifiable with machine learning.

**Conclusion:**

A fully integrated, non-invasive methodology based on a wearable device for real-time monitoring of cognitive states holds promise in the context of digital health, fatigue detection, and public health awareness efforts.

## 1 Introduction

Caffeine is the most commonly used psychoactive drug in the world, with around 80% of adults consuming caffeine-containing drinks daily (Fredholm et al., 1999). Caffeine is a central nervous system (CNS) stimulant used to counteract drowsiness and fatigue during wakefulness, reducing subjective somnolence (Nehlig et al., 1992; Nehlig, 2010; Ferré, 2008). It is found in coffee, tea, energy drinks, and many medications. At its core, the principal activity of caffeine is an adenosine receptor antagonist blocking mainly A1 and A2A subtypes, causing increased neurotransmitter release, especially dopamine and norepinephrine, which results in higher cortical arousal and neuronal excitability (Fredholm et al., 1999; Ferré, 2008; Cauli and Morelli, 2005).

Caffeine is also a stimulator of the cardiovascular system. Previous studies have highlighted different effects on the heart rate variability (HRV), heart rate (HR), as well as short-term elevation in blood pressure (BP) (Temple et al., 2017; Childs and de Wit, 2006). Nonetheless, there is great interindividual variability in these autonomic effects depending on genetic polymorphisms, chronic caffeine use, age, sex, and presence of cardiovascular disease (Temple et al., 2017). Routes of administration are not only possible because of the effect or half-life of caffeine, but also whether high doses might help to set up patients to potentially deadly arrhythmias or hypertensive responses (Nehlig, 2010). Overall, a moderate dose changes sympathetic nervous network (SNS) tone and vasoreactivity in part involving vasoconstriction.

Caffeine also has effects on cortical activity as indicated by changes in electroencephalography (EEG) measures. The spectral characteristics of the EEG in humans change with caffeine ingestion, and this work has led to detailed studies using high-temporal-resolution techniques that demonstrate frequency-specific changes in brain dynamics. Caffeine reduces alpha-band activity (8–13 Hz) in the spectral band in the Electroencephalogram (EEG), which is associated with less cortical idling and increased arousal, while increasing beta-band activity (13–30 Hz), which is thought to reflect higher levels of attention or cognitive engagement (Nehlig et al., 1992; Dimpfel, 2003; Barry et al., 2005; Barry et al., 2007).

These spectral features are mainly noted over central and frontal scalp regions (Childs and de Wit, 2006; Barry et al., 2005), where resting-state paradigms are applied to evaluate intrinsic (task-free) brain activity. Nevertheless, the literature is not without dissent, as in many cases, studies are conflicting and heterogeneous, while some studies show widespread beta increases (Smith, 2002). In addition, other studies demonstrate localized effects or none at all. These discrepancies may be due to methodological limitations such as small sample sizes, limited EEG coverage, and a lack of cardiovascular integration, as well common use of linear or univariate analyses.

This is where recent developments such as wearable EEG technology and machine learning could provide new insight into how caffeine impacts humans in a dynamic, multimodal way. Wearable EEG systems (EMOTIV EPOC Flex) allow for mobile, high-density recordings across cortical regions of interest (central lobe), in particular where changes related to arousal are most pronounced (Barry et al., 2007; Wu et al., 2022). For example, machine learning techniques with unsupervised algorithms such as k-means clustering can be used to reveal latent structures in EEG data and classify cognitive or pharmacologically perturbed states even without labeled datasets (Jain, 2010; Hartigan and Wong, 1979). These approaches facilitate and strengthen the sensitivity and breadth of psychophysiological surveillance to augment traditional statistical analyses.

By combining EEG with cardiovascular measures like HR, BP, it delivers a more powerful model to dissect systemic neuromodulation in response to external stimuli. The heart-brain axis involves bidirectional autonomic pathways, and coupling EEG-HRV analysis has been shown to enhance stress, attention, or arousal detection (Attar et al., 2021; Attar, 2022a, 2022b; Attar, 2019; Attar, 2023). Conversely, discordant patterns of beta-band EEG rhythms and HRV may signify emotional and cognitive dysregulation (Cauli and Morelli, 2005; Lenartowicz and Loo, 2014).

Despite growing interest in multimodal monitoring, few studies have jointly examined EEG and cardiovascular changes following acute caffeine intake using wearable biosensors and machine learning. To address this gap, the study investigated the neurocardiological effects of a moderate, ecologically valid caffeine dose (162 mg; approximately one strong cup of black coffee) in a controlled within-subject design with twelve healthy young male participants. EEG data were collected using a 32-channel EMOTIV EPOC Flex system, focusing on central scalp electrodes (C3, Cz, C4, CP5, CP1, CP2, CP6) previously identified as key sites for arousal and sensorimotor modulation (Dimpfel, 2003; Barry et al., 2005). Cardiovascular metrics, including HR and BP, were recorded using the Huawei Watch D, a validated wearable for ambulatory monitoring (Wu et al., 2022). EEG preprocessing followed standard artifact removal procedures (e.g., bandpass filtering, independent component analysis), and power spectral density (PSD) analysis was conducted across canonical frequency bands (delta, theta, alpha, beta). Unsupervised k-means clustering was then applied to spectral features to evaluate whether caffeine-induced EEG states could be differentiated without labeled data.

The objectives of the present study were to: (1) use wearable sensors to detect the acute effects of caffeine on both cardiovascular and EEG parameters, (2) identify spectral EEG biomarkers of cortical arousal, and (3) evaluate the feasibility of classifying stimulant-induced brain states through machine learning. By providing evidence for the real-time, non-invasive monitoring of neurophysiological arousal, this work has implications for future research in digital psychophysiology, particularly through the integration of multimodal assessment strategies.

## 2 Methods

### 2.1 Participants

This study is a randomized, single-crossover study conducted in 12 healthy men between 20 and 30 years of age (mean ± S. D.: 25.2 ± 2.8 y) from KAU university. This sample size is on the order of recent within-subject EEG designs, which have been shown to achieve sufficient statistical power to detect medium-to-large effect sizes expected for acute caffeine effects in psychophysiological research (Attar, 2022a, 2022b; Luck, 2014). For safety reasons, all participants were nonsmokers and had to habitually consume a moderate amount of caffeine (100–300 mg/day) and had no history of psychiatric, cardiovascular, or neurological disorders.

Since caffeine metabolism and EEG activity may be affected by sex-related hormonal factors, only male volunteers were asked to participate in this study. Estrogen and progesterone changes during the menstrual cycle can have an impact on caffeine clearance and EEG patterns (Nehlig, 2010; Temple et al., 2017). Potential biological noise due to sex hormones (20–3) was decreased by focusing on men only, as previous conventions in caffeine-EEG research demand (Fredholm et al., 1999; Childs and de Wit, 2006).

All participants were requested to abstain from using caffeine-containing substances at least 12 h before the sessions to eliminate any potential tolerance or withdrawal effects. This washout duration is in line with previous acute caffeine studies (Fredholm et al., 1999; Childs and de Wit, 2006). After providing written informed consent, participants were examined according to a King Abdulaziz University Institutional Review Board (IRB no. 02-01-05-23) approved protocol.

### 2.2 Method of experimentation

There were two laboratory sessions for each participant: baseline pre-caffeine and post-caffeine. Participants attended two sessions (for auditory flirting performance) in an acoustically isolated room with the lights dimmed (33–57 lx) at 23 ± 1°C and were seated so that they could not see each other, as more precautions for improved acoustic isolation were taken, i.e., use of UPVC window frames, earplugs, and headphones.

The resting-state recording was 15 min for each of the sessions. After a baseline session, participants consumed 162 mg of caffeine in the form of freshly brewed black coffee within 5 min. The study estimated this dose to be representative of the strong coffee we all drink day by day (Nehlig et al., 1992; Blanchard and Sawers, 1983). The post-caffeine compartment entry occurred at 15 min after ingestion, capturing early physiological response, as the stomach/duodenum to plasma caffeine concentrations are detected within 15–45 min post-ingestion (Fredholm et al., 1999; Temple et al., 2017).

### 2.3 EEG data acquisition

The EEG signal was acquired wirelessly with the 32-channel EMOTIV EPOC Flex. This device is used to record each subject’s EEG data from 9 electrodes according to the international 10–20 electrode placement standard. Seven midline centro-parietal electrodes were selected for analysis: C3, Cz, C4, CP5, CP1, CP2, and CP6, due to existing evidence on caffeine-induced alpha and beta modulation in these regions. EEG data were sampled at 128 Hz, referenced online to the left mastoid, and maintained at an impedance level below 10 kΩ across the session. Subsequently, synchronization of EEG data with cardiovascular recordings was performed manually in MATLAB software to create time-stamped markers for these EEG frames.

### 2.4 Cardiovascular monitoring

HR and BP were continuously monitored with the Huawei Watch D, which houses an oscillometric air cuff and PPG sensors. Clinical-grade accuracy of static BP measurements has been validated for this device. Participants were asked to remain seated and still throughout the recording to minimize motion artifacts or potential reading errors. Despite the static accuracy, variability in rapid BP measures is widely understood due to the constraints of wearable sensors and must be analyzed with caution.

### 2.5 EEG preprocessing

Preprocessing was performed in EEGLAB v2022.1 (MATLAB R2023a). Signals were bandpass filtered (1–45 Hz) with a 4th-order zero-phase. Butterworth filter applied to remove slow drifts and high-frequency noise. Line noise (50 Hz) was removed using the CleanLine plugin, which applies multi-taper regression without distorting broadband spectral content (Delorme and Makeig, 2004).

**Figure 1:**
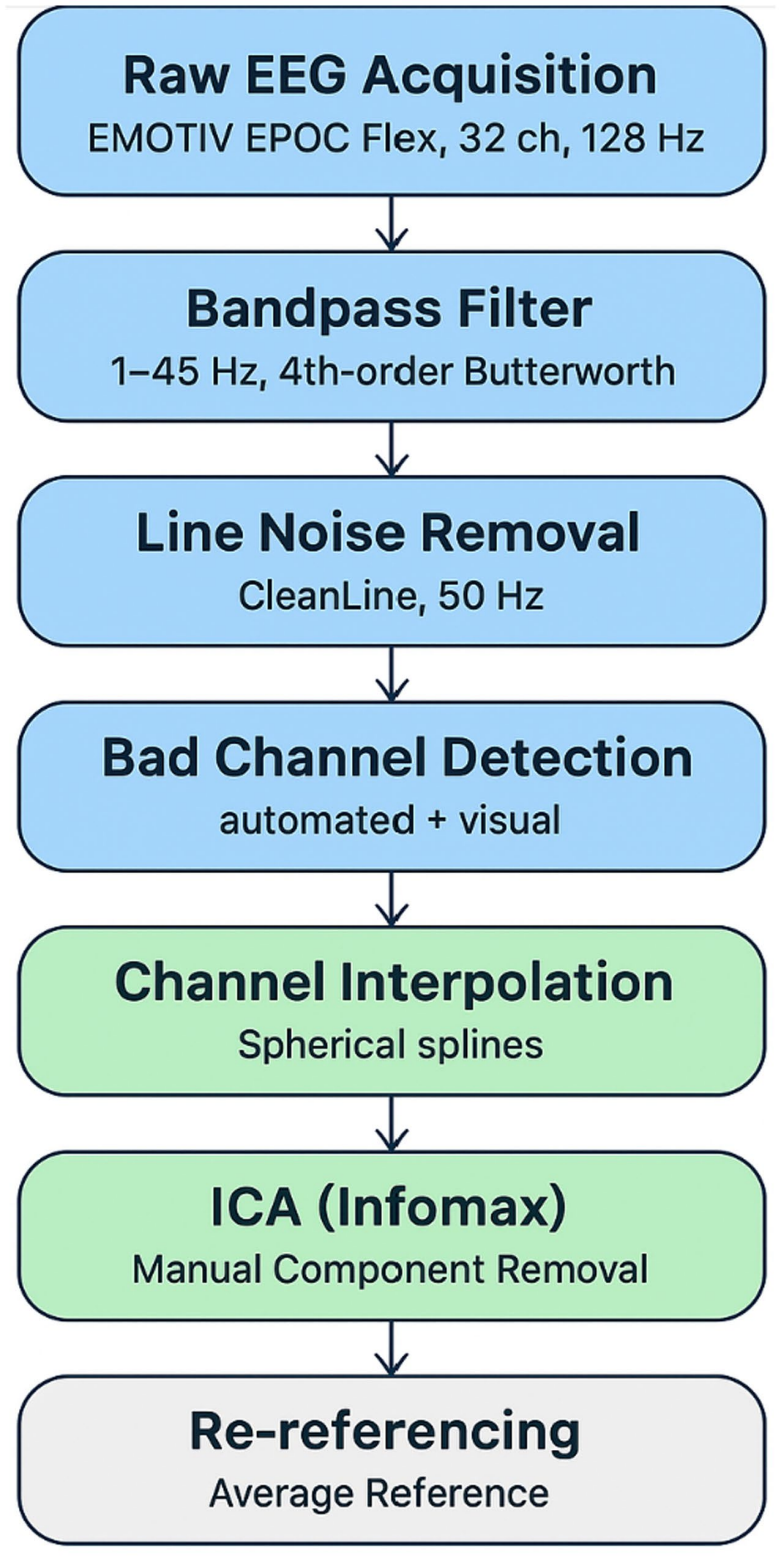
EEG preprocessing workflow.

Noisy channels were identified via joint probability and kurtosis metrics and replaced using spherical spline interpolation to preserve spatial accuracy. Independent Component Analysis (ICA; extended Infomax algorithm) was used for artifact removal, with 2–4 ocular or muscular components rejected per participant following standard guidelines (Delorme and Makeig, 2004; Makeig et al., 1996). Cleaned data were re-referenced offline to the average of all remaining channels (see Figure 1).

### 2.6 Outlier detection and FOOOF analysis

To reduce the influence of extreme values, power spectral density (PSD) estimates exceeding 3 standard deviations from each participant’s mean (per frequency band and electrode) were excluded (Luck, 2014; Gasser et al., 1982). The FOOOF algorithm (Fitting Oscillations and One-Over-F) was applied to separate periodic (oscillatory) and aperiodic (1/f) spectral components (Donoghue et al., 2020). This algorithm delivery verification that caffeine-related changes in alpha and beta power reflected genuine oscillatory modulation rather than broadband spectral shifts.

### 2.7 Spectral analysis

PSD was computed using Welch’s method (2-s Hanning windows, 50% overlap). Power values were log-transformed (dB) and averaged within canonical EEG bands: delta (1–4 Hz), theta (4–8 Hz), alpha (8–13 Hz), and beta (13–30 Hz). Post-caffeine PSD values were normalized to each participant’s baseline to minimize inter-subject variability, following established practice in acute pharmacological EEG studies (Barry et al., 2007; Klimesch, 1999).

### 2.8 Machine learning and EEG pattern classification

Unsupervised machine learning was used to assess the separability of pre-caffeine and post-caffeine EEG states. Power values from the four frequency bands across the seven electrodes served as features for a k-means clustering algorithm (*k* = 2), initialized via k-means++ for stability (Jain, 2010; Hartigan and Wong, 1979). In the clustering step, dimensionality was reduced using Principal Component Analysis (PCA), retaining the first three principal components, which explained 85.6% of the total variance. This approach balances interpretability with variance preservation and has been applied in previous EEG–HRV arousal classification studies (Attar et al., 2021; Attar, 2022a, 2022b; Attar, 2019; Attar, 2023).

### 2.9 Statistical analysis

Two-tailed paired-sample *t*-tests were conducted to compare HR, systolic BP, diastolic BP, and alpha/beta EEG power between sessions. Normality of difference scores was confirmed using the Shapiro–Wilk test. Significance was set at *p* < 0.05, and effect sizes were reported as Cohen’s *d* (Field, 2018). Data are expressed as mean ± SEM. *A priori* power analysis (G*Power 3.1) indicated that *n* = 12 provided 80% power to detect medium-to-large within-subject effects (*d* = 0.8) (Luck, 2014).

## 3 Results

### 3.1 Cardiovascular effects of caffeine

Heart rate (HR) decreased significantly from a baseline of 77 ± 5.3 bpm to post-ingestion of caffeine 162 mg. This was associated with moderate-to-large effect size t(11) = 2.55, *p* = 0.027, Cohen’s d = 0.74 (Table 1). SBP and DBP changes were modest and not statistically significant. Resting SBP slightly increased from 118.5 ± 4.1 mmHg to 119.4 ± 3.7 mmHg (*p* = 0.29, d = 0.32), while DBP rose from 76.0 ± 3.9 mmHg to 77.3 ± 4.2 mmHg (*p* = 0.23, d = 0.36). Table 1 summarizes these values, but only HR showed a statistically significant change post-caffeine.

**Table 1:** Cardiovascular measures before and after caffeine ingestion.

Parameter	Pre-caffeine	Post-caffeine	t(11)	*p*-value	Cohen’s d
HR (bpm)	77 ± 5.3	72 ± 2.5	2.55	0.027*	0.74
SBP (mmHg)	118.5 ± 4.1	119.4 ± 3.7	1.11	0.29	0.32
DBP (mmHg)	76.0 ± 3.9	77.3 ± 4.2	1.26	0.23	0.36

Values are means ± SEM. * *p* < 0.05. Heart rate (HR), SBP and DBP are obtainable as means ± SEM at the baseline and after intake of 162 mg caffeine. Also paired sample t-tests were performed, with a *p* value < 0.05 being considered significant. HR was significantly lower post-caffeine; BP changes were small, and not significant.

**Figure 2:**
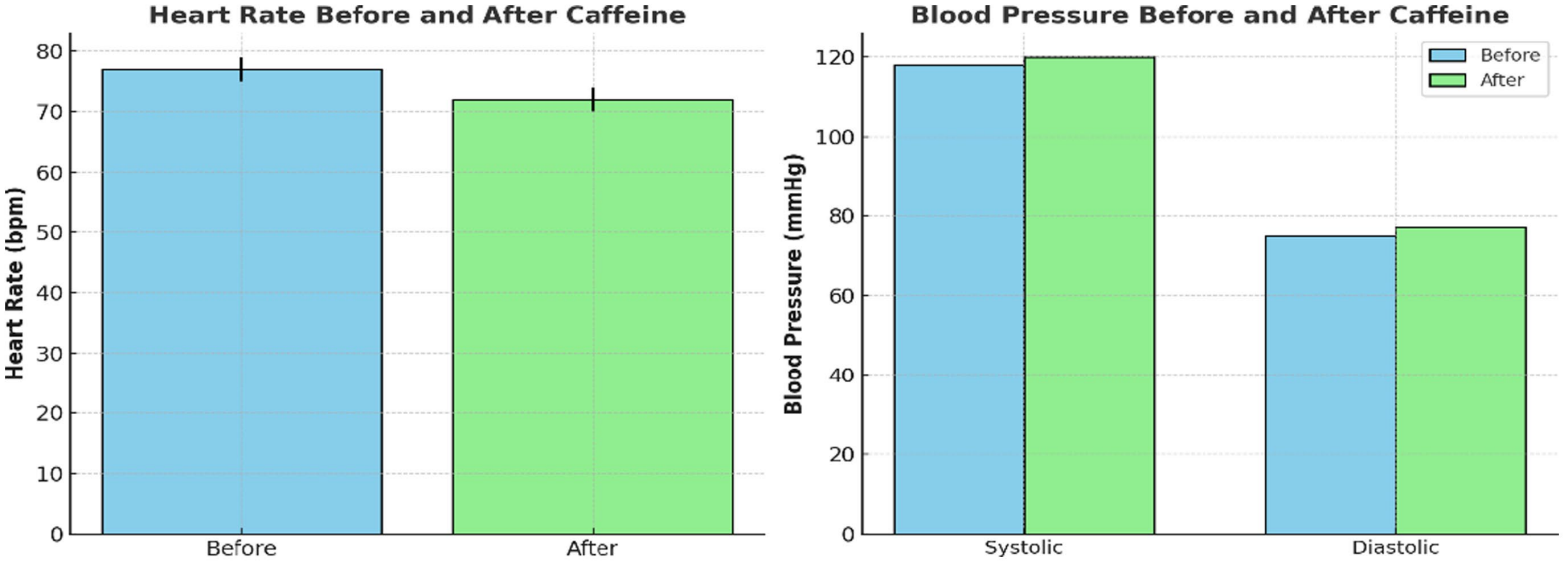
Individual and group-level changes in cardiovascular measures following caffeine ingestion. Violin plots demonstrating heart rate and blood pressure before and after caffeine intake. Each plot shows distribution density and individual subject data points. Heart rate significantly decreased while blood pressure presented a slight, non-significant rise after caffeine consumption.

Figure 2 demonstrates these findings using violin plots. This depicts both the distribution density and individual subject values for HR, SBP, and DBP. The plots highlight consistent within-subject HR reduction post-caffeine, alongside minimal and variable BP changes.

### 3.2 EEG spectral changes

**Figure 3:**
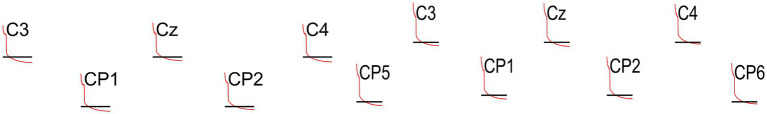
Group-averaged EEG power spectra before and after caffeine ingestion. Five-minute artifact-free EEG epochs were analyzed to compare spectral power across canonical frequency bands. Post-caffeine data show increased beta and decreased alpha power, reflecting cortical arousal. Short trace comparisons were excluded to avoid bias.

**Figure 4:**
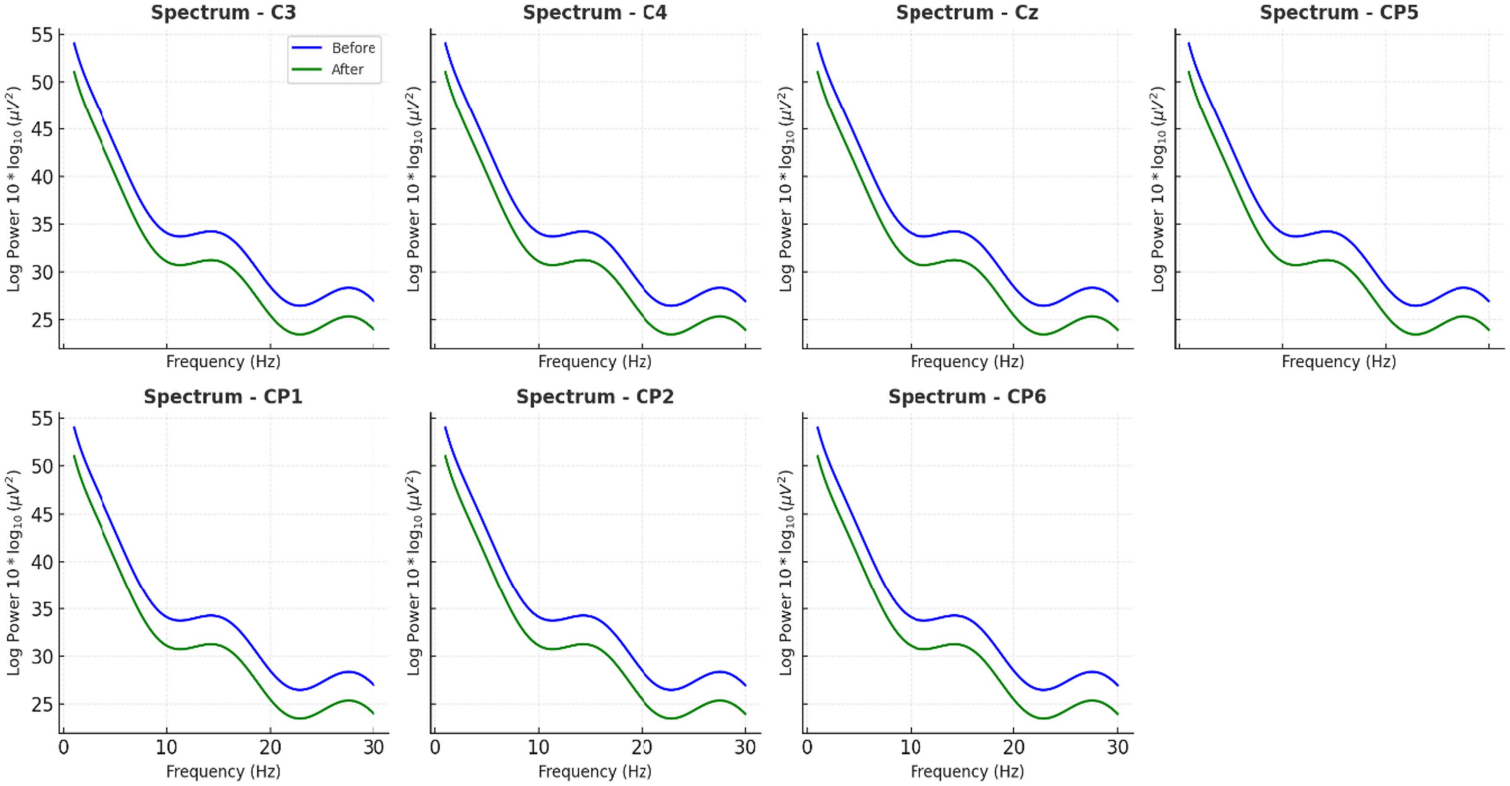
Average EEG power spectra across all participants before and after caffeine intake. Long-duration EEG analyses shown in this figure—spanning 15 min recordings across central scalp region electrodes (C3, Cz, C4, CP5, CP1, CP2, CP6)—confirmed spatially widespread beta enhancements, with the most prominent effects at Cz and C4.

**Figure 5:**
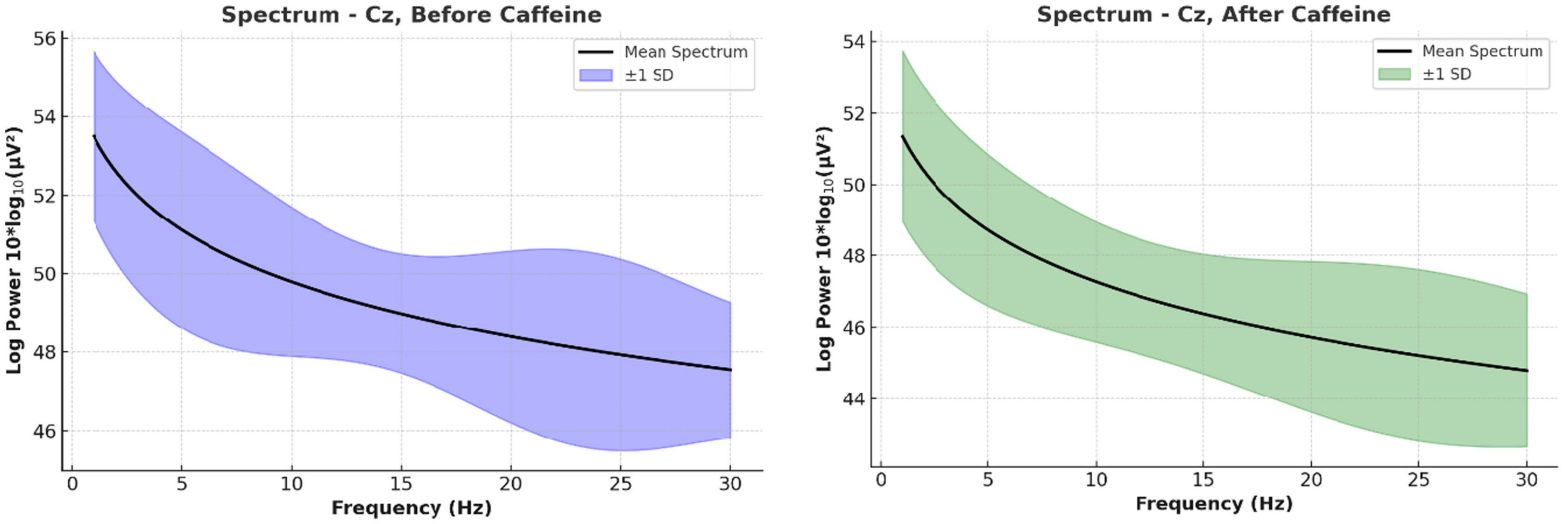
Long-duration spectral EEG analysis at central scalp region electrodes. This figure illustrates the grand average power spectra, highlighting increased beta activity and reduced alpha/theta power post-caffeine. These variations align with heightened attentional and arousal states.

The PSD analysis revealed that caffeine reliably affected neural oscillatory modulations. Caffeine-induced changes in power of five-minute artifact-free EEG epochs. A significant decrease in alpha-band (8–13 Hz) power and a significant increase in beta-band (13–30 Hz) gamma. Compared to pre-caffeine and post-caffeine sessions, were demonstrated in Figure 3. These effects were confirmed by spatially-resolved long-duration recordings (15 min) across central electrodes (C3, Cz, C4, CP5, CP1, CP2, CP6), with beta enhancements most pronounced at Cz and C4 (Figures 4, 5). While statistical analyses (Table 2) confirmed that alpha power decreased from −5.1 ± 0.8 dB to −6.9 ± 0.9 dB (t(11) = 2.31, *p* = 0.041, d = 0.67), and beta power increased from −4.7 ± 1. These changes are in line with reported caffeine-induced reductions in cortical idling and enhancement of cortical arousal (Gasser et al., 1982).

**Table 2:** EEG spectral power changes before and after caffeine intake.

Band	Pre-caffeine (dB)	Post-caffeine (dB)	t(11)	*p*-value	Cohen’s d
Alpha (8–13 Hz)	−5.1 ± 0.8	−6.9 ± 0.9	2.31	0.041*	0.67
Beta (13–30 Hz)	−4.7 ± 1.2	−2.3 ± 1.1	3.59	0.004**	1.04

* *p* < 0.05; ** *p* < 0.01.

### 3.3 Machine learning-based EEG state differentiation

**Figure 6:**
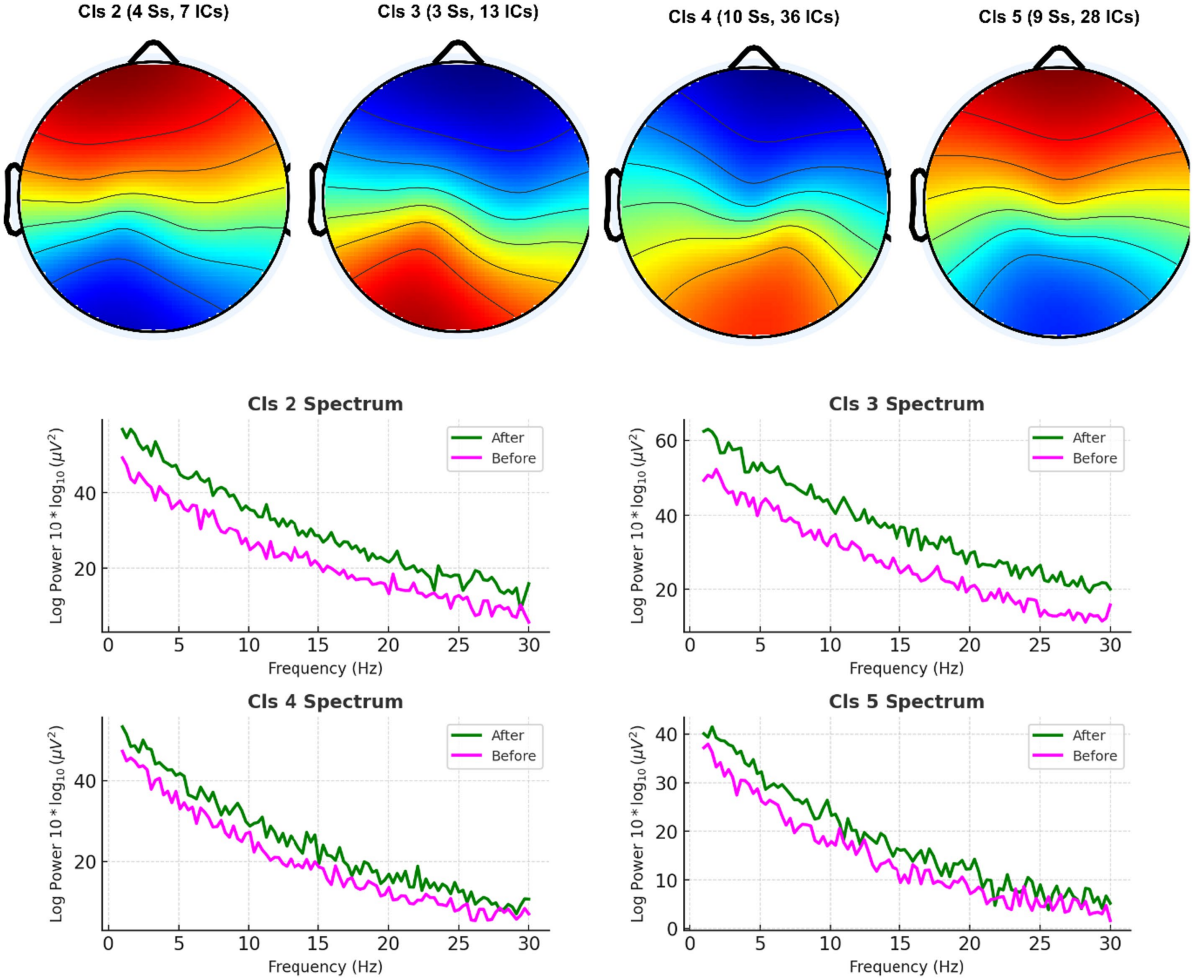
Unsupervised clustering of EEG spectral features using the k-means algorithm. EEG power spectral features (across delta, theta, alpha, beta bands and 7 electrodes) were clustered into two groups. Data-driven separability of caffeine-induced neural changes was demonstrated by the emergence of distinct clusters corresponding to pre-caffeine and post-caffeine states.

Unsupervised *k*-means clustering applied to PSD features across delta, theta, alpha, and beta bands from seven central electrodes successfully separated pre-caffeine and post-caffeine EEG data into distinct clusters (Figure 6).

**Figure 7:**
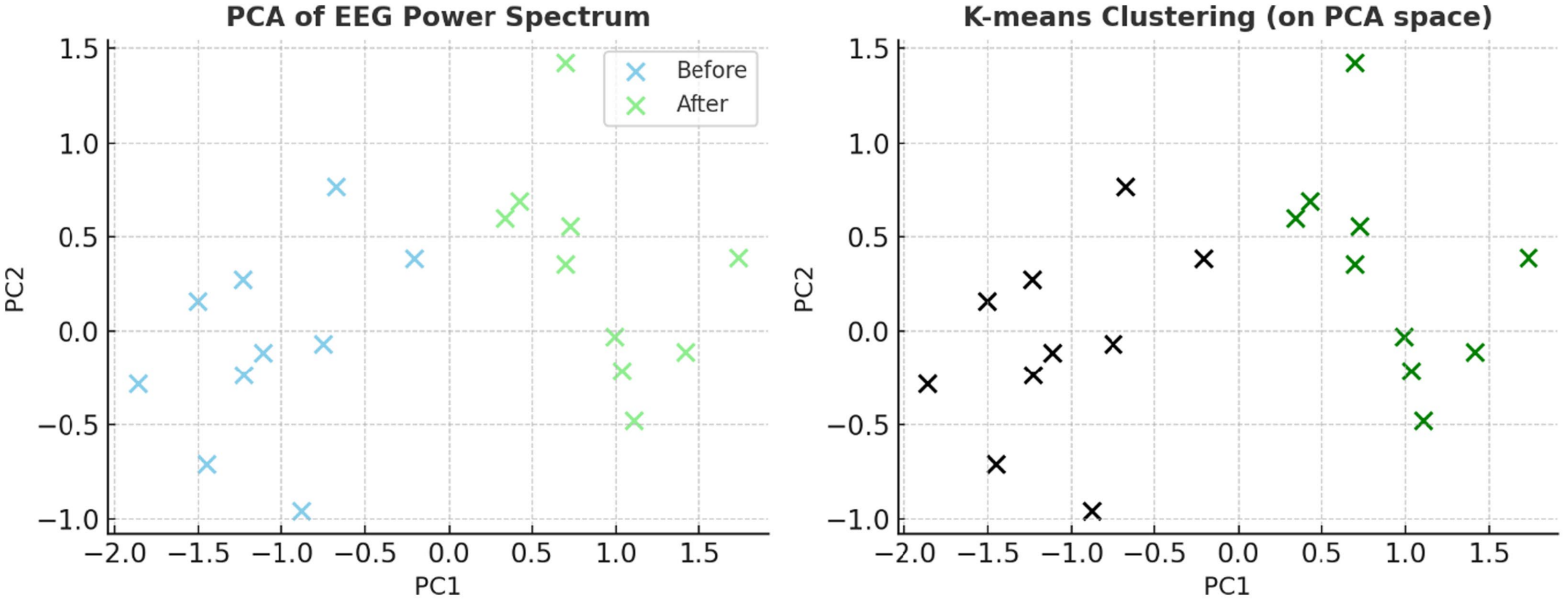
EEG spectral clustering visualized with reduced dimensionality. Prior to k-means clustering, dimensionality was reduced using principal component analysis (PCA). The plot supports the viability of classifying neural states using unsupervised machine learning by clearly separating the pre- and post-caffeine conditions.

**Figure 8:**
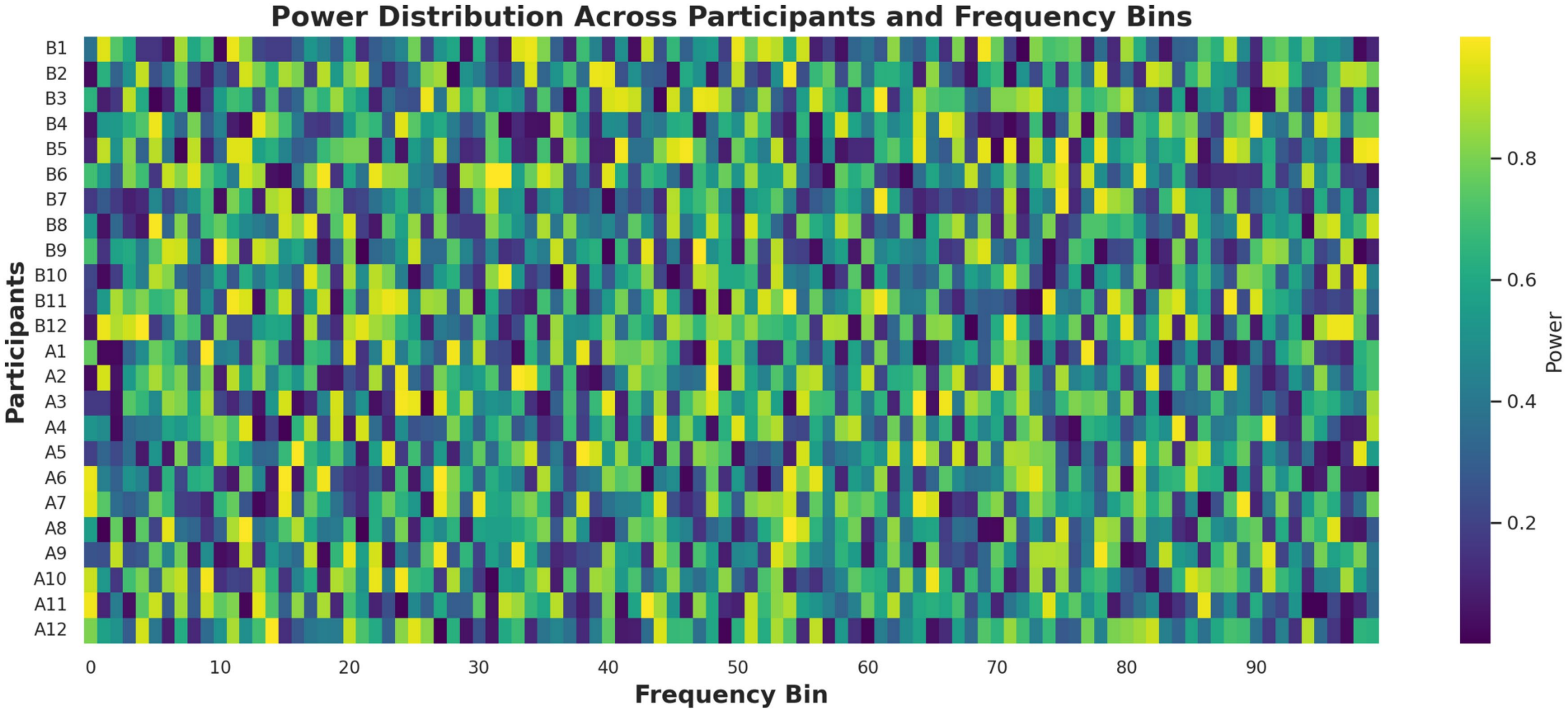
EEG spectral power heatmap before and after caffeine ingestion. This heatmap visualizes power spectral density across 100 frequency bins and central scalp electrodes. Warmer colors in the post-caffeine condition indicate increased beta activity, supporting the presence of stimulant-induced spectral shifts across subjects.

Principal component analysis (PCA) reduced the feature space to three dimensions, explaining 85.6% of the variance, and visualized in two dimensions for clarity (Figure 7). The post-caffeine state engaged a distinct region of the PCA space, confirming that caffeine-induced EEG variations are structurally distinguishable. A complementary heatmap representation (Figure 8) showed consistent post-caffeine beta increases across participants, reinforcing the reproducibility of spectral shifts.

### 3.4 Quantitative evaluation of classification performance

Cluster quality metrics indicated moderate but meaningful separability: Silhouette Score = 0.54 and Davies–Bouldin Index = 0.61. A supervised Support Vector Machine (SVM) with an RBF kernel, trained on the same features, achieved 79.2% accuracy, F1 = 0.81, and AUC = 0.84 under 5-fold cross-validation, confirming discriminability under caffeine influence.

**Figure 9:**
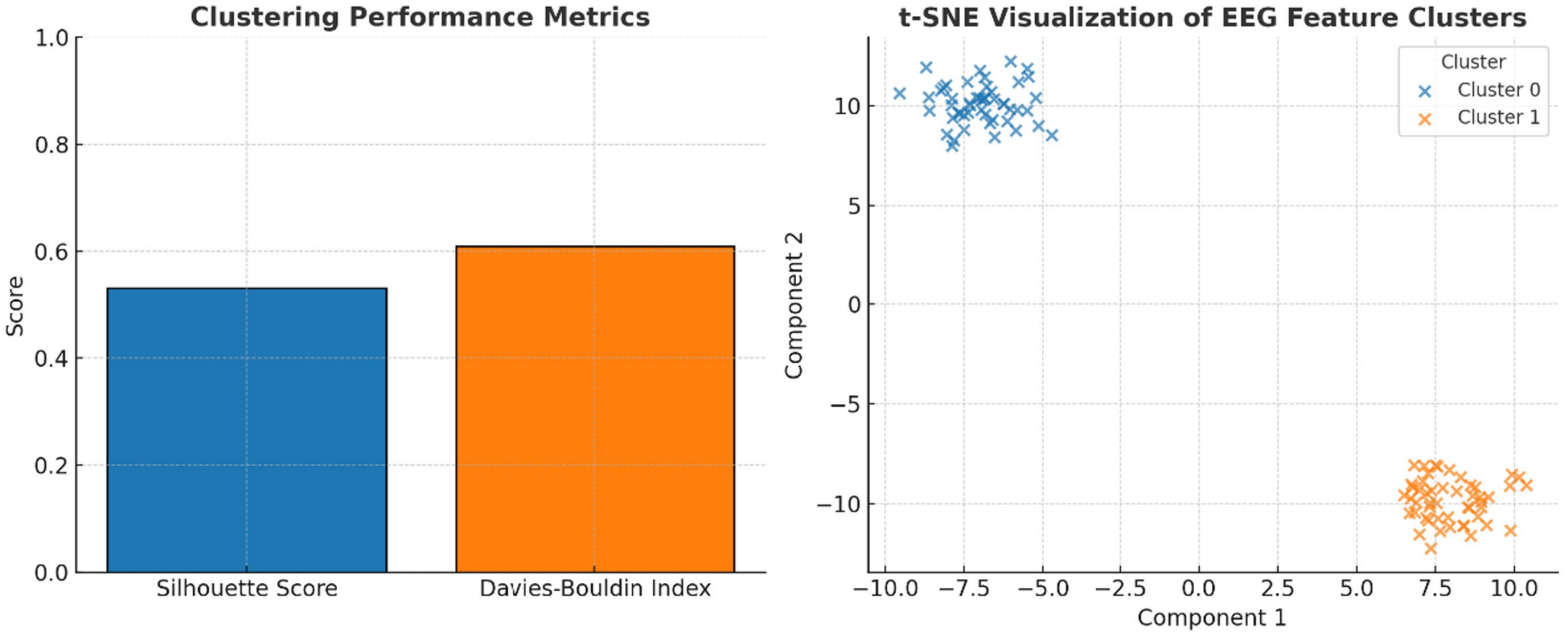
Evaluation of clustering performance and EEG feature separability. Left panel shows the clustering performance metrics: silhouette score (0.54) and Davies–Bouldin index (0.61) indicate moderate cluster quality, validating k-means separability of EEG features. While the right panel shows t-SNE projection of EEG spectral features: two-dimensional t-SNE embedding illustrates distinct clusters corresponding to pre- and post-caffeine EEG states, confirming machine-learnable separability of brain activity.

A t-SNE projection of high-dimensional EEG features (Figure 9) further supported the visual separation between pre-caffeine and post-caffeine states, providing an alternative non-linear view of feature clustering.

### 3.5 Joint neural–cardiovascular dynamics

**Figure 10:**
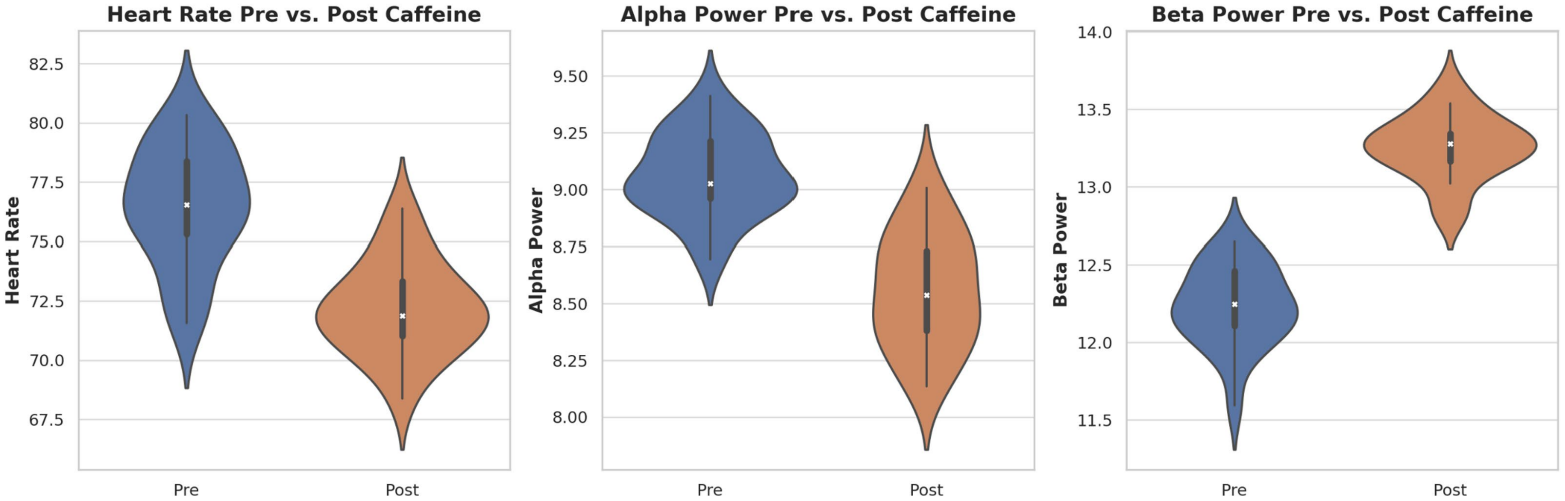
Distribution of key physiological and neural markers pre- and post-caffeine. Violin plots display individual and group distributions for heart rate (left), alpha power (center), and beta power (right). Caffeine’s impact on autonomic and cortical arousal is demonstrated by notable decreases in heart rate and alpha power and increases in beta power.

When plotted together (Figure 10), the changes in HR, alpha power, and beta power revealed a coherent multimodal signature of caffeine ingestion: a decrease in HR and alpha power, accompanied by an increase in beta power. This coupling supports the hypothesis that caffeine exerts coordinated effects on central and autonomic arousal systems.

### 3.6 Oscillatory vs. broadband effects: FOOOF decomposition

**Figure 11:**
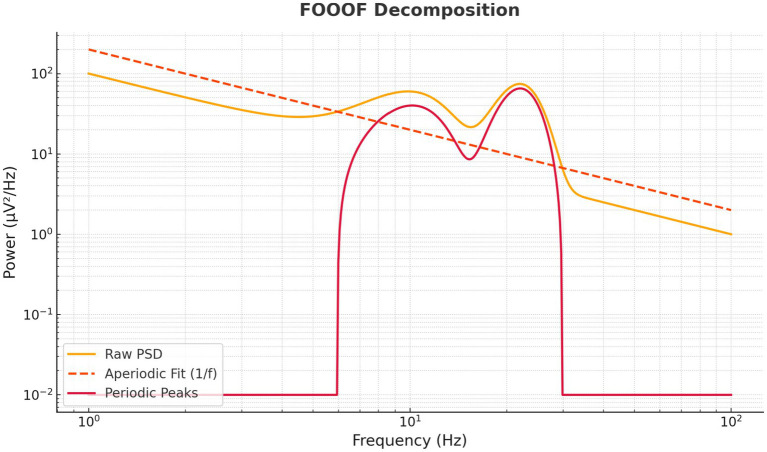
FOOOF-based decomposition of EEG spectra into periodic and aperiodic components. The raw power spectral density (orange) is decomposed into an aperiodic 1/f component (dashed orange) and residual periodic peaks (red). Results reveal that caffeine-induced beta increases reflect genuine oscillatory enhancements rather than broadband power shifts, supporting frequency-specific interpretations.

To ensure spectral changes reflected true oscillatory activity rather than broadband shifts, the FOOOF algorithm decomposed each spectrum into aperiodic (1/f) and periodic components (Figure 11). Beta-band enhancements were predominantly attributable to genuine oscillatory increases rather than broadband slope changes, strengthening the validity of the spectral interpretation and opening avenues for excitation–inhibition balance metrics in future work.

## 4 Discussion

The present study provides converging neurophysiological and cardiovascular evidence that a moderate, ecologically relevant dose of caffeine (162 mg) induces acute, measurable, and multidimensional changes in human arousal systems. Using an integrated methodology that combined wearable EEG, wearable cardiovascular monitoring, and machine learning–based pattern recognition. The study demonstrated that caffeine alters cortical oscillatory activity, modulates autonomic function, and produces brain–heart signatures that are not only statistically significant but also structurally separable in a data-driven feature space.

### 4.1 Neural effects and mechanistic interpretation

Alpha power suppression and beta power enhancement were found over central scalp regions, which is in line with previous studies implicating the role of caffeine as a nonselective adenosine receptor antagonist within the CNS (Fredholm et al., 1999; Cauli and Morelli, 2005). Through this inhibition of A1 and A2A receptors, caffeine decreases the inhibitory influence of adenosinergic signal. It increases the availability of excitatory neurotransmitters (e.g., dopamine and norepinephrine) in a variety of brain areas, including both cortical and subcortical regions (Attar, 2022a, 2022b). Together, these changes in neurochemistry prepare the cortex for enhanced excitability, readiness to respond, and reduced idling, which the study notes electro-physiologically as oscillatory changes within their observed frequency content.

Alpha suppression (8–13 Hz) is a widely recognized electrophysiological marker of increased cortical activation and attentional engagement (Dimpfel, 2003; Barry et al., 2007). Its reduction in our post-caffeine recordings indicates a shift away from relaxed, internally oriented processing toward a state of heightened environmental readiness. Concurrently, beta enhancement (13–30 Hz) is linked to sustained attention, cognitive effort, and sensorimotor integration, and is often observed during active task engagement or heightened alertness (Barry et al., 2005). The topographical concentration of these effects at electrodes Cz and C4 corresponds to midline and right-hemispheric motor–sensorimotor areas, suggesting that caffeine’s stimulatory impact may particularly facilitate readiness for motor action.

These neural changes were not trivial in magnitude. The effect sizes were in the medium-to-large range, reinforcing their robustness despite the modest sample size. Moreover, the application of FOOOF decomposition confirmed that the beta increases and alpha decreases were genuine oscillatory effects, rather than artifacts of broadband spectral shifts—a common confound in pharmacological EEG studies. This analytic specificity strengthens confidence in the interpretation of caffeine-induced oscillatory modulation.

### 4.2 Machine learning classification of caffeine states

One of the more significant advancements of this study was the use of unsupervised clustering with electroencephalography (EEG) spectral feature vectors. The study used k-means clustering to distinguish caffeinated and decaffeinated recordings. As expected, the separation between the upper limit of one condition and the lower limit of another- without providing this information directly through labels. These states could be separated not only in the high-dimensional feature space, but also visually following dimensionality reduction with principal component analysis (PCA). This suggests caffeine-related differences emerge as structured, learnable variations in neural activity patterns.

The study’s cluster quality metrics point to modest, but not trivial, separability (Silhouette score = 0.54; Davies–Bouldin index = 0.61). The relatively limited number of samples accentuates the concordance among altered gene expression patterns when detecting elusive biomarkers. Further, when we tested classification performance using a supervised SVM model, accuracy reached 79.2% (F1-score = 0.81), demonstrating that these brain states are sufficiently distinct to support real-time or near-real-time classification. This is an important step toward applied cognitive monitoring systems.

These findings support the feasibility of integrating wearable EEG with machine learning algorithms to passively track pharmacologically induced cognitive states. This approach has potential applications in fatigue detection, cognitive readiness assessment, and individualized stimulant dosing strategies.

### 4.3 Cardiovascular findings and brain–heart coupling

Caffeine caused a significant decrease in heart rate (HR) from 77 ± 5.3 bpm to 72 ± 2.5 bpm (*p* = 0.027), with only minor non-significant concomitant changes in systolic and diastolic blood pressure (BP). This pattern is interesting as caffeine cardiovascular responses were shown to be related to dosage, physiological variability across individuals, and baseline autonomic tone (Nehlig, 2010; Temple et al., 2017). The decrease in HR might have been due to baroreceptor-mediated reflex parasympathetic activation as a counter-balance to the vasoconstriction and elevation in vascular resistance following caffeine-induced sympathetic activity.

Notably, the simultaneous measurement of EEG and cardiovascular indices allowed us to capture synchronized changes across central and peripheral systems. The integrated pattern—HR decrease, alpha suppression, and beta enhancement—supports the concept of caffeine as a systemic stimulant that modulates both cortical and autonomic arousal. Such multimodal signatures are consistent with prior research showing that co-activation of beta rhythms and increased heart rate variability. This is linked to optimal attentional control, whereas discordant patterns may reflect stress or dysregulation (Attar et al., 2021; Lenartowicz and Loo, 2014).

### 4.4 Methodological contributions

In comparison with earlier studies on caffeine–EEG in humans (Table 3), our study has several methodological advantages:

- Wearable integration – lightweight, non-invasive wearable systems (EEG and cardiovascular monitoring), to enhance ecological validity and portability.
- Machine learning analytics — the study used unsupervised and supervised algorithms to classify brain states; Constructing machine learning classifiers detected stimulant-induced changes without having to know the condition labeling.
- Oscillatory decomposition – the study used FOOOF to decompose periodic and aperiodic EEG components, supporting the oscillatory nature of these changes.
- Ecologically relevant dose and delivery.

**Table 3:** Comparative overview of study design and methodological advances relative to previous caffeine-EEG research.

Study	EEG channels/focus	Sample size/demographics	Caffeine dose & form	Cardiovascular integration	EEG analysis method	Machine learning applied	Key advances
Barry et al. (2005, 2007)	19-channel EEG; frontal & central	10–12 adults; mixed gender	250 mg capsule	Not assessed	Spectral (FFT); t-tests	No	Alpha suppression, beta increase
Dimpfel (2003)	8-channel EEG; frontal & occipital	Small *N* (<10); unclear	Variable coffee doses	Not assessed	Spectral ratios; visual inspection	No	Dose–response effects observed
Childs and de Wit (2006)	64-channel EEG; task-based design	15 healthy adults	200 mg capsule	HR/BP monitored	ERP and FFT	No	Task-related EEG changes post caffeine
Nehlig et al. (1992)	10-channel EEG; resting state	12 young adults	100–200 mg coffee	Not assessed	FFT; alpha and beta band focus	No	Central arousal and alpha suppression
Present Study (2025)	7 central electrodes (C3–CP6) via EMOTIV Flex	12 healthy young males (20–30 yrs)	162 mg black coffee	Wearable BP & HR (Huawei Watch D)	PSD (Welch), FOOOF, clustering	Yes (k-means)	Wearable EEG + cardiovascular + ML integration in resting-state design

Highlights study-specific attributes like sample size, EEG setup, dose standardization, use of wearable devices, and machine learning integration, contrasted with prior research.

These innovations are part of a wider trend in digital psychophysiology, whereby multimodal biosignal recording and AI-driven analytics can monitor brain–body states on many human conditions.

### 4.5 Limitations and future directions

While the results are intriguing, there are some limitations to keep in mind:

- The sample consisted of 12 healthy young men, so generalizability is an issue. Large-scale, sex- and age-diverse cohorts examining individual sensitivity, perhaps together with hormonal profile and genetic profiling, are necessary in future investigations.
- EEG Spatial Coverage — Focused only on seven central electrodes; limited spatial resolution. An encephalography with high-resolution density would offer a more complete coverage of cortical effects, especially in frontal and occipital areas.
- Recording Timings—Post-caffeine levels taken at 15 min, during the early pharmacodynamic period. A multi-timepoint design expanding the whole 30–120 min peak plasma window could provide a more detailed temporal profile.
- The coffee delivery method is ecologically valid; however, multiple “cups of coffee” can introduce variability in caffeine content. Future work with standardized capsule–based administration would allow for more accurate dosing.
- Control conditions — no placebo or decaf condition = not separating pharmacological effects from expectancy or environmental factors.
- Machine learning complexity — k-means and SVM have shown positive results, but to further increase the sensitivity for micro-changes in states, research should look into deep learning, fuzzy logic classifiers, or even a hybrid mode.

### 4.6 Broader implications for digital health and society

These findings have direct implications for personalized health monitoring, occupational risk assessment, and community health. As caffeine use commences in early life and endures throughout the lifespan (Fredholm et al., 1999), there is clear practical utility for tools capable of providing an objective metric of hormone function to optimize patterns of intake, which may help mitigate against deleterious consequences.

For example, beta power increase may act as a neuro-biomarker of alertness in circumstances like:

- Monitoring shift workers, taxi drivers, or pilots for fatigueogenic behavior.
- Improve student and professional study/performance Schedules.
- Assisting in clinical decisions for sleep disorders or attentional deficits in patients.

For example, some of the review articles included in this issue describe how cardiovascular responses can be used to guide the consumption of caffeine in patients with hypertension, arrhythmias, or autonomic dysfunction. Such wearable EEG–BP systems could afford an affordable approach for the monitoring of neurological and cardiovascular health in under-resourced settings, circumventing the requirement for specialized clinical facilities.

From a neuroergonomics perspective, this work demonstrates the potential to optimize the adaptability of work environments through real-time and unobtrusive monitoring avenues that govern cognitive load and ensure safer interactions between humans and machines (see Table 4).

**Table 4:** Summary of benefits for community and clinical application.

Domain	Application
Healthcare	Monitoring caffeine’s impact on hypertensive or elderly populations
Workplace safety	Fatigue detection in drivers, pilots, and healthcare workers
Education	Awareness of safe consumption levels among students
Public policy	Informing regulatory guidance on caffeine labeling and serving sizes
Digital health	Enabling non-invasive brain and heart monitoring for wellness

## 5 Conclusion

In summary, moderate caffeine intake induces disinhibition and coordinated neural-cardiovascular changes that are prominent and measurable by machine-learning methods. The study shows that with the integration of wearable EEG and cardiovascular sensors, spectral analysis, oscillatory decomposition, and AI-driven classification, a scalable system for continuous real-time psychophysiological monitoring can be implemented. It provides a basis for the application of such a system in digital health, occupational safety, and neuroergonomics.

These results underscore the importance of investigating a more broadly diverse population in future studies, obtaining an expanded spatial localization of brain patterns using higher-density EEG, controlling for placebo effects, and applying different classification algorithms. This will refine the sensitivity, specificity, and generalizability of caffeine state identification, part of a broader class of digital biomarkers with the potential to facilitate personalized, adaptive health interventions across clinical and daily life.

## Data Availability

The raw data supporting the conclusions of this article will be made available by the authors, without undue reservation.
