# Correction: Caffeine on the mind: EEG and cardiovascular signatures of cortical arousal revealed by wearable sensors and machine learning—a pilot study on a male group

**DOI:** 10.3389/fnsys.2025.1708544

**Published:** 2025-10-07

**Authors:** Shabbir Chowdhury, Ahmed Munis Alanazi, Eyad Talal Attar

**Affiliations:** ^1^Department of Electrical and Computer Engineering, Faculty of Engineering, King Abdulaziz University, Jeddah, Saudi Arabia; ^2^Center of Excellence in Intelligent Engineering Systems (CEIES), King Abdulaziz University, Jeddah, Saudi Arabia

**Keywords:** caffeine, EEG, heart rate variability, blood pressure, cortical arousal, beta waves, machine learning, wearable sensors

In the published article, in the **Funding** section, an incorrect **Funding** statement was erroneously provided. Instead of the incorrect grant number “(IPP:948-135-2025)”, it should have been written as “(IPP:941-135-2025)”. The corrected Funding statement appears below:

