# Supplementary figures and images for: Caffeine on the mind: EEG and cardiovascular signatures of cortical arousal revealed by wearable sensors and machine learning—a pilot study on a male group
Source: Front Syst Neurosci. 2025 Sep 15;19:1611293. doi: 10.3389/fnsys.2025.1611293 (PMC12477153; doi:10.3389/fnsys.2025.1611293)

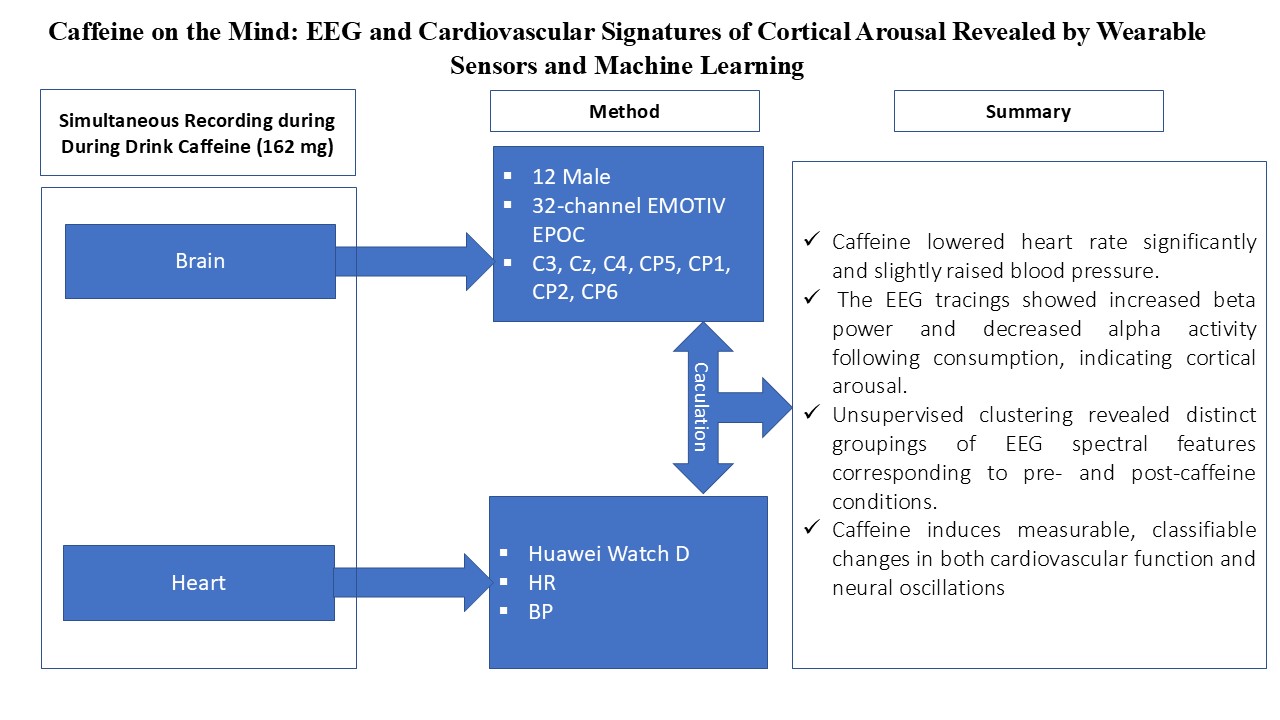

Supplement: Supplementary file 1 [file Image_1.JPEG]
